# Risk Factors and Behaviours of Schoolchildren with Myopia in Taiwan

**DOI:** 10.3390/ijerph17061967

**Published:** 2020-03-17

**Authors:** Han-Chih Cheng, Koyin Chang, Elizabeth Shen, Kai-Shin Luo, Yung-Hsiang Ying

**Affiliations:** 1 Department of Ophthalmology, Taipei Tzu-chi Hospital, New Taipei City 231, Taiwan; hanchihcheng@gmail.com (H.-C.C.); lizshen88@gmail.com (E.S.); seekcoeur@gmail.com (K.-S.L.); 2 Department of Ophthalmology, Tzu-chi University, Huanlien County 907, Taiwan; 3 Dept. of Healthcare Information and Management, Ming Chuan University, Taoyuan City 333, Taiwan; 4 Department of Ophthalmology, National Taiwan University Hospital, Medical College, National Taiwan University, Taipei 100, Taiwan; 5 Department of Business Administration, National Taiwan Normal University, Taipei 106, Taiwan

**Keywords:** myopia progression, environmental factors, vision care knowledge

## Abstract

*Importance:* Because of the high prevalence of myopia in Taiwan, understanding the risk factors for its development and progression is important to public health. *Background:* This study investigated the risk factors for myopia and their influence on the progression of myopia in schoolchildren in Taiwan. *Design:* Patients’ clinical records were obtained retrospectively from ophthalmologists. Questionnaires were given to collect demographic information, family background, hours spent on daily activities, myopia progression, and treatment methods. *Participants:* From a regional medical hospital in northern Taiwan, 522 schoolchildren with myopia participated in the study. Written informed consent was obtained from participants of legal age or the parents or legal guardians of younger children. *Methods:* Multivariable regression analyses were performed. Myopia measured in cycloplegic spherical equivalent (SE) was analysed, controlling for patients’ family and demographic information as well as their daily activity behaviours. *Main Outcome Results:* Children with high myopic parents were more myopic. Earlier onset age of myopia was associated with a higher level of myopia and greater annual myopic progression. Children reporting longer time usage of electronic devices had greater progression of myopia. Boys tended to be more myopic than girls. Lower levels of myopia were associated with more outdoor activities, and better vision care knowledge in children and parents. *Conclusions and Relevance:* In addition to genetics, education and environment can influence the development of myopia. Health policies for schoolchildren should promote protective activities and vision care knowledge at a young age, to protect the eyesight of schoolchildren.

## 1. Introduction

Myopia is a highly prevalent disease throughout the world. High myopia is often associated with vision debilitating diseases such as cataracts, glaucoma, maculopathy, and retinal detachment [1,2,3]. Myopia-associated maculopathy is reported as a leading cause of irreversible blindness in many countries [4,5,6,7,8,9]. Previous research determined that controlling myopic progression in children could lower the relative risk of development of myopic maculopathy, retinal detachment, and posterior subcapsular cataract by about 5 times, 3 times, and 1.5 times, respectively [10]. Therefore, screening for myopia in children and preventing or reducing myopic progression are of paramount importance in protecting and promoting public health.

About 30 years ago, the Taiwan Ministry of Education (MOE) initiated the Taiwan Student Vision Care Program (TSVCP) to combat myopia. Nationwide, school nurses conduct regular vision testing in elementary schools every semester. Students with vision below 20/25 are required to visit an ophthalmologist for further evaluation [11]. This national screening program has resulted in approximately 80% of schoolchildren in Taiwan being seen by ophthalmologists for further eye care and intervention to control myopia progression. Despite these efforts, myopia prevalence in Taiwanese schoolchildren did not decrease significantly. Therefore, understanding possible environmental and lifestyle factors that may contribute to myopia progression is essential in promoting the ocular health of these children.

In this study, we investigated the environmental and lifestyle factors associated with myopia progression in schoolchildren regularly followed at our outpatient clinic in a regional medical hospital in Northern Taiwan. A specially designed questionnaire was employed to collect the socioeconomic background and daily activity information of the subjects, along with the clinical data obtained from the ophthalmologists’ office. Data were then submitted for multivariate regression analyses, with a focus on the impact of daily activities controlling socioeconomic factors, food supplements, and background knowledge about eye care. The results of this study may identify the potential risk factors for myopia, which may influence future health policies for vision care in children.

## 2. Methods

### 2.1. Study Design and Patient Population

To understand the risk factors for progression of myopia and the influence of each factor, a specially designed survey of schoolchildren’s family backgrounds and daily activities was conducted. Following conventional modern medical observational studies, this research adopts a questionnaire that relies on self-reported subjective answers responded by the schoolchildren or their parents/legal guardians [12,13,14]. The questionnaire combined patient characteristics and behaviour with clinical information. The study targeted those who, at the time of our study, were either referred to ophthalmologists’ offices after failing the school vision screening or were returning myopia patients of the ophthalmologists. Only schoolchildren aged 6–20 years were included in the study. The data collection period was from February 2018 to November 2018. All the patients were referred to the interviewers by their ophthalmologist after full explanation of the purpose of the study and obtaining written informed consent from the participants (if of legal age) or their parents or guardians. This study was carried out according to the tenets of the Declaration of Helsinki and the Research Governance Framework for Health and Social Care. The questionnaire was completed by parents/guardians and schoolchildren together, or by the children themselves if they were old enough to read the questions.

Of the 603 patients invited to take part, 522 completely answered all of the questions. The inclusion criteria for our subjects were: (i) outpatients with myopia or pseudomyopia; (ii) speakers of Chinese or Taiwanese; (iii) age between 6 and 20 years; (iv) ability of parents/guardians to provide informed consent; and (v) lack of other serious eye diseases. In this study, myopia is defined as a cycloplegic spherical equivalent (SE) less than −0.5 diopters (D) in the worse eye. The cycloplegic refraction is measured after using 1 gtt 0.4% tropicamide (Mydrin-M) every 5 min, 3 times, and then the refractive error is measured 30 min after the first drop of tropicamide, by both autorefraction and retinoscopy [15]. Pseudomyopia is defined as a noncycloplegic SE less than −0.5D, but a cycloplegic SE greater than −0.5D in the worse eye. Since there is a strong correlation between the fellow eye of a subject in terms of myopia progression, this study used data from one eye of each subject [16].

### 2.2. Ethics, Governance, and Consent

Clinical data were obtained from patients’ ophthalmologists regarding their refractive error measured in dioptres, myopia progress since the last annual visit, and treatment. To include appropriate questions in the survey, the potential risk factors that contribute to myopia were drawn from a systematic review of the relevant literature [11,17,18,19]. To ensure that the research does not cause social, legal or economic risks to the subjects, this study was approved by the Institutional Review Board of Taipei Tzu-Chi Hospital (identifier, 06-X22-086) for potential ethical issues.

### 2.3. Outcome Measures

Two types of outcome measures were obtained. Firstly, the degree of myopia was measured with cycloplegic refraction for both eyes, which was measured in dioptres [20]. The eye with greater myopia was selected for analysis. Secondly, the information of progress of myopia was collected retrospectively by calculating the decrease in the schoolchildren’s SE in the past year. Children of all ages are measured in the same way, and the technique used in this study is consistent across ophthalmologists in Taiwan.

### 2.4. Risk Factors

The myopia risk factors were obtained from the recent literature [17,18,19,21,22]. Shih et al. reported the effectiveness of atropine on myopia progression in Taiwanese schoolchildren [23]. Thus, whether or not the myopia was treated with long-acting mydriatics/cycloplegics (i.e., atropine 0.125%) was considered as an influential factor. Genetics has long been linked directly to myopia. Thus, parents’ myopia or not, self-reported by respondents, was included as an effective measure of the genetic factor. Daily activities, such as hours spent on outdoor activities, sleep, study (near work activity), TV watching, usage of electronic devices with an illuminated screen, and hours spent in “cram school” (confined environment) after regular school hours, may potentially affect children’s vision. Family background information, such as living space, income level, and parents’ knowledge about vision care, was controlled for in the analyses. Clinical information included the presence of other eye problems such as amblyopia or heterotropia, intake of food supplements, academic achievement measured by school grade, height, weight, age of onset of myopia (the age of diagnosis of myopia), frequency of office visits, and the use of vision corrective tools.

### 2.5. Vision Care Knowledge

Schoolchildren’s vision can be better cared for if parents have the relevant vision care education. Ten questions, designed in a 5-point Likert scale, were used to ascertain the eye care knowledge of the parents/guardians (or the students if of legal age). A score of 5 on the Likert scale meant strongly agree and 1 meant strongly disagree. The questions were as follows.

- Long-acting mydriatic agent is harmful to eyes.
- Long-acting mydriatic agent is proven to be effective in controlling myopia.
- Vision deteriorates faster for people with myopic squinting and seeing without appropriate corrective lenses.
- It is better not to wear eyeglasses, even when you have myopia, since eyeglasses may very likely make your vision worse.
- Pupil dilating effect will disappear once you stop using mydriatic agents.
- Corrective lens power should be less than your real power since the full correction lenses may accelerate myopia progression.
- Usage of electronic devices will accelerate myopia progression.
- Eye health and vision can be improved by adequate outdoor activities and exercise.
- “30/10 Rule” refers to every 30 min spent in near work activity requires a 10-min rest.
- High myopia is nothing to worry about. It is not associated with retinal problems, glaucoma, cataracts, or any other eye diseases.

For the above ten questions, a higher number in the Likert scale meant better knowledge, except for the four questions that were reverse-scored (a, d, f, and j). Based on the answers, each respondent received a knowledge index score from 1 to 100, with higher scores indicating a greater level of vision care knowledge.

### 2.6. Sampling

Myopia is a common eye problem in Taiwan. Since the Taiwan Ministry of Education (MOE) and Ministry of Health and Welfare have collectively implemented an eye care program in all levels of schools and the vision impairment referral rate has achieved 80%, it is reasonable to believe that outpatient services for myopia cover a representative range of the schoolchildren with myopia. The patients surveyed were from Tzu-Chi Hospital, Taipei Branch, which is a regional medical hospital in northern Taipei.

### 2.7. Multivariate Regression Analysis

The ordinary least square (OLS) method was employed to explain the variation in degree of the schoolchildren’s myopia as well as their myopia progression. A refractive error measurement for each child was obtained. The progression of myopia in the past year was transcribed from the children’s medical records to gain an understanding of the extent to which each risk factor contributed to the progression of myopia. Various functional forms of OLS estimates were explored to find a good model of fit, including linear, natural log, and quadratic forms. This study focuses on the impact of schoolchildren’s daily activity behaviors controlling socioeconomic, genetic, environmental, and nutritional factors. To conduct a multivariate regression method with unbiased results, any possible factors that contribute to variation in the dependent variable are expected to be included in the regression model, to avoid potential omitted variable problems [24,25]. Our regression procedure follows conventional socioeconomic studies for observational analyses. Similar studies can be found in the extant literature [13,14,26].

## 3. Results

### 3.1. Patient Demographics

Table 1 provides the descriptive analysis of the schoolchildren’s demographic information, including age, gender, family income, and the distribution of degree of myopia. Of all the sampled schoolchildren, the average age (± standard deviation) was 11.31 (±3.08) years; boys comprised 49.81% of the schoolchildren with myopia. On average, participants had a follow-up visit to the ophthalmologist office every 4.17 (±1.80) months. Approximately 22.56% of the sampled patients went to private school, which is considered to be more demanding in terms of behavioral discipline. A total of 62.45% of the sampled schoolchildren also attended an independent private class after school for test preparation or extended learning, or so-called “cram schools” in Taiwan. Their daily activities included an average daily habit of using electronic devices, TV watching, studying, and engaging in outdoor activities of 1.94 (±1.43), 1.77 (±1.16), 3.08 (±1.10), and 3.68 (±3.42) hours, respectively. The average age of myopia onset was 8.24 (±2.71) years. Columns (2) to (4) present the results by educational stages. The progression of myopia within the past year is most severe for primary schoolchildren and least for high school students.

Table 2 presents the schoolchildren’s intake of food supplements and treatment for myopia. The intake of vitamins, fish oil, and lutein among schoolchildren was 15.32%, 9.34%, and 13.98%, respectively. In our sample, 97.31% of the schoolchildren used mydriatics/cycloplegics, with 28.8% using a long-acting mydriatic agent Atropine. Among our sample of 522 schoolchildren, 57.28% used vision correction devices such as spectacles, contact lenses, or orthokeratology.

Table 3 lists the perceived causes of myopia of the schoolchildren, based on the results of the survey. According to the literature, extensive usage of the eyes for reading/studying and TV watching are important behavioural-related causes of myopia [21,27,28]. A lack of outdoor activities and sleep are also shown to contribute to an increase in refractive error [17,29,30]. The sampled children in this study reported that a lack of outdoor activities was most likely to cause their myopia, followed by extensive hours of reading, and TV watching, with approval rates of 3.80 (±1.20), 3.71(±1.18), and 3.57(±1.35), respectively, on the 5-point Likert scale.

### 3.2. Multivariate Analyses and Sensitivity Tests

In order to understand the extent to which each factor contributes to schoolchildren’s myopia, multivariate regression analyses were conducted. The absolute value of the refractive error measurement (D) was employed as the dependent variable. Various functional forms of OLS estimates were explored to find a good model of fit, including linear, natural log, and quadratic forms. All forms show similar results and only the log form is reported for the reasons of convenience in results interpretation, and to conserve space. The analysis results, as shown in Table 4, were drawn from three regression estimations with models (1), (2), and (3) representing the total sample, primary schoolchildren, and secondary schoolchildren, respectively. The results suggest that children with a younger age at onset (the number of years since being diagnosed) tended to have a higher degree of myopia. Children or parents with better vision care knowledge and children with better school academic performance tended to have less severity of myopia. The results were significant at the 1% or 5% significance level. Children going to private schools and families concerned about their living or activity spaces tended to have milder cases of myopia. The use of long-acting mydriatics was positively related to more severe myopia. One or both parents being myopic was positively related to children’s severity of myopia. Boys were more likely to have a higher degree of myopia, especially boys in primary school. The intake of vitamins was positively related to myopia severity in primary school, but negatively related in secondary school. The intake of other food supplements, however, was not significantly related to the severity of myopia.

In terms of daily activities, time spent studying/reading did not significantly increase the level of myopia for children. The effect was even negative for primary schoolchildren (*p* < 0.1). Children who spent more time in outdoor activities had lower levels of myopia. Spending more hours in cram school tended to increase the myopia level (*p* < 0.1). These effects, however, were not significant when estimated separately by school level. Usage of electronic devices did not result in a higher degree of myopia.

To understand the factors that contribute to the progression of myopia, further regression analyses were performed, employing the change in refractive error measurement (in absolute value) in the past year of the sampled children. The results are presented in Table 5. Number of years with myopia again played an important role in myopia progression (*p* < 0.1 and *p* < 0.05 for the all sample and primary school sample, respectively). Growth in height and BMI were also positively related to increases in myopia (*p* < 0.05). Schoolchildren’s weight was negatively related to myopia progression, especially for children in primary school. Regarding daily activities, the time spent using electronics was positively associated with myopia progression (*p* < 0.05 and *p* < 0.1 for the all sample and secondary school sample, respectively). No other activities showed significant effects.

**Figure 1:**
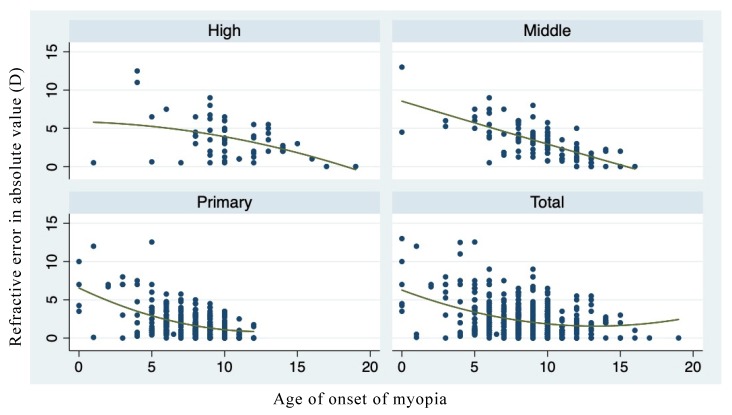
Age of onset of myopia and current myopia severity of the schoolchildren.

**Figure 2:**
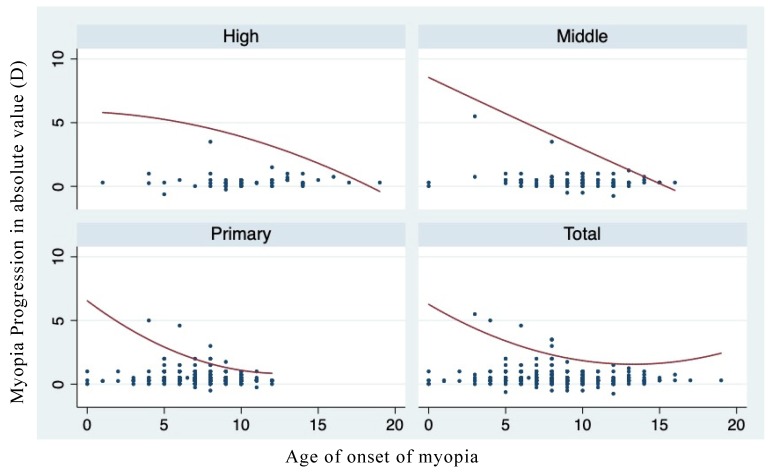
Age of onset of myopia and the myopia progress in the past year.

Figure 1 shows the relationship between the age of onset of myopia and the current myopic severity of schoolchildren at different educational stages. Children with an earlier onset age of myopia had higher current levels of myopia. Focusing on myopic progression in the past year, we see a similar relationship between the age of onset of myopia and the myopic progression in the past year, as shown in Figure 2. Children with an earlier onset age of myopia had more myopic progression in the past year.

## 4. Discussion

Myopia has long been a public health issue in many developed countries. Understanding the risk factors and eye care principles related to myopia are important for promoting both the eyesight and the general health of the population as a whole, since myopia is linked to many serious eye complications. In our study, only 57.28% of subjects use corrective methods, leaving 43% not using anything. This ratio may seem high at first glance. Given the average refractive error −2.42D in our sample, many children are considered mild cases of myopia, and they could still see clearly in the classrooms. Thus, parents may not deem it to be urgent to have spectacles prescribed for their children. Parents sometimes even believe that wearing the lenses more often may develop the habit of dependence, which in turn would aggravate the problem. Thus, the low ratio is reasonable in our sample.

The results of our study are, in general, consistent with the extant research in terms of genetic factors and the impact of outdoor activities, near work activities, and usage of screen devices [17,30,31,32]. Further implications can be drawn from our findings, as follows. First, the intake of food supplements did not significantly impact on schoolchildren’s myopia progression. One may argue that the impact of food supplements can be bi-directional, since patients with more severe myopia may want to use more food supplements. With annual sales of $567 million in the food supplement market in Taiwan, of which approximately 50.85% is for eye care [33], it is noteworthy that food supplements to prevent or control myopia may have little effect in the short term. Further research on the impact of food supplements is crucially needed. Second, people believe that extensive reading/studying and a lack of outdoor activities are the most important risk factors for myopia. However, when people are aware of this possibility and concerned that their living conditions are not spacious enough, they take precautions to prevent myopia and have better eyesight than those who are not concerned (*p* < 0.05). Third, better eye care knowledge in parents resulted in children with better eyesight (*p* < 0.05). Fourth, the time spent using electronic devices was positively related to the progression of myopia and this result was especially true for secondary school students (*p* < 0.05). Fifth, academic performance was negatively related to myopia severity for primary schoolchildren (*p* < 0.01). This effect vanished for secondary schoolchildren. Previous studies mostly indicate a positive relationship between myopia and academic achievement, using genetic linkage and environmental factors to explain the phenomena [34]. For younger children, school performance does not require many hours of study and near work activities, suggesting that the previous findings of a positive relationship might be more related to environmental than to genetic factors. Lastly, it is important to build up good habits in eye protection for children in elementary school. Even though high school students are considered to use their eyes more intensively than younger children for studying or using electronic devices, their myopia progression was not as fast as that of younger children, implying that the onset of myopia at an earlier age is especially harmful for one’s vision. Therefore, delaying the onset age of myopia may significantly reduce its severity.

### Limitations

Our participants were urban schoolchildren with different levels of myopia. The results may not be applicable to students who live in rural areas with a different lifestyle. No children with perfect eyesight are included in the study. The comparison basis lacks this group of schoolchildren. Cycloplegics/mydriatics, such as atropine or tropicamide, have been the mainstream treatment of myopia in Taiwan since the late 1990s [23]. It is not ethical if we do not treat our myopic patients with cycloplegic agents. Thus, over 97% of our subjects use cycloplegic agents in this study. The high percentage of cycloplegics treatment among our subjects is another limitation of our study. In addition, no information on comorbidities or healthcare service utilization related to other diseases was incorporated in the study. Since eye conditions can be related to other health issues, missing this information could be a potential shortcoming in this research.

## 5. Conclusions

Previous research has extensively discussed the risk factors for myopia and emphasizes the significant impact on the severity of myopia and its progression of genetic factors, school achievement, and daily activities such as reading/studying and outdoor activities. In this study, the genetic factor continues to show significant impact on myopia severity, consistent with the findings of the existing studies [18]. Other findings of this study suggest that behaviors, such as near work activities and environmental factors, are influential in myopia pathogenesis [35]. The length of time staying in cram school was positively associated with the level of myopia as well as myopia progression. Early onset age of myopia was especially detrimental. Parent- or self-knowledge of myopia prevention, and self-awareness of being in a confined environment, were related to less severity of myopia. The findings suggest that policies that promote eye care education, the awareness of environmental influences on myopia, and the importance of outdoor activities starting from a young age are effective measures to protect the eyes of schoolchildren and help them maintain better eyesight throughout their lives.

## Figures and Tables

**Table 1:** Subject demographic information.

	Total	Primary	Middle	High School
**Number**	522	344	115	63
**Age (years)**	11.31 (3.08)	9.58 (1.91)	13.55 (1.21)	16.60 (1.32)
**Boy (%)**	49.81	53.20	45.22	39.68
**BMI ^†^**	18.63 (4.02)	17.57 (3.48)	20.08 (4.01)	21.89 (4.31)
**Family Income (USD, monthly)**	3132.32 (1382.7)	3180.3 (1394.15)	3084.93 (1339.60)	2885.20 (1394.26)
**Frequency of seeing an opthalmologist (months)**	4.17 (1.80)	3.92 (1.80)	4.62 (1.65)	4.76 (1.87)
**Private School (%)**	22.56	19.82	23.00	36.51
**Cram School (%)**	62.45	64.50	66.37	44.44
**Activity Hours**				
**Cram School (hours per day)**	1.98 (1.97)	2.17 (2.06)	1.81 (1.83)	1.11 (1.49)
**Electronic Devises (hours per day)**	1.94 (1.43)	1.54 (1.26)	2.30 (1.36)	3.43 (1.27)
**TV (hours per day)**	1.77 (1.16)	1.83 (1.10)	1.63 (1.15)	1.71 (1.41)
**Study (hours per day)**	3.08 (1.10)	2.93 (0.97)	3.26 (1.21)	3.50 (1.24)
**Sleep (hours per day)**	7.66 (1.12)	7.90 (1.11)	7.29 (0.95)	7.00 (0.96)
**Outdoor activities (hours per week)**	3.68 (3.42)	3.80 (3.19)	3.65 (4.15)	3.16 (3.19)
**Refractive error (D) ^‡^**	2.42 (2.13)	1.95 (1.83)	3.20 (2.23)	3.62 (2.54)
**Myopia Progression (D) (in past year)**	0.50 (0.63)	0.53 (0.61)	0.47 (0.74)	0.42 (0.56)
**Knowledge**	76.52 (10.22)	77.18 (9.96)	75.00 (10.51)	75.67 (10.92)
**Onset Age**	8.24 (2.71)	7.46 (0.18)	9.46 (2.95)	10.36 (3.20)
**Other Eye Disorders**				
**Amblyopia**	9.41%	9.55%	8.03%	11.11%
**Strabismus**	5.69%	6.27%	5.36%	3.17%

Numbers in parentheses represent ± standard deviation. ^†^ Body Mass Index (BMI) is calculated as body mass (in kg) divided by the square of body height (in m) or kg/m^2^. ^‡^ Myopia or myopia progression are represented as the absolute value of the spherical equivalent (SE) in dioptres (D). Onset Age is the age at which the schoolchildren were diagnosed with myopia. USD, US dollars. Activities are presented as hours per day except for outdoor activities, which is reported as hours per week. Knowledge is an index range from 1 to 100, a higher score represents better vision care knowledge of the parents.

**Table 2:** Intake of food supplements for eye care and treatment for myopia.

	**Total**	**Primary**	**Middle**	High School
**Number**	522	344	115	63
**Supplements (%)**				
**Vitamin**	15.32	14.82	19.13	11.11
**Fish oil**	9.34	10.50	7.82	6.35
**Lutein**	13.98	13.08	14.78	17.46
**Mydriatic (%)**	97.31	97.09	97.39	98.41
**Atropine (0.125%)**	28.8	31.39	21.73	23.80
**Corrective methods (%)**	57.28	49.72	72.17	71.42
**Spectacles**	56.32	48.54	71.30	71.42
**Contact lenses**	3.25	2.48	5.21	4.76
**Orthokeratology**	0.57	0.00	1.73	1.58

Note: Mydriatic includes short-acting agents and long-acting ones; the former is 0.4% Tropicamide (Mydrin-M) and the latter is 0.125% Atropine.

**Table 3:** Perceived causes of myopia.

	Total	Primary	Middle	High School
**Reading**	3.71 (1.18)	3.74 (1.21)	3.65 (1.10)	3.63 (1.15)
**TV**	3.57 (1.35)	3.67 (1.32)	3.27 (1.49)	3.61 (1.26)
**Outdoor activities**	3.80 (1.20)	3.86 (1.17)	3.61 (1.27)	3.78 (1.14)
**Sleep**	3.45 (1.31)	3.36 (1.31)	3.48 (1.37)	3.85 (1.10)
**Genetics**	3.45 (1.26)	3.54 (1.26)	3.44 (1.22)	3.00 (1.20)
**Space**	2.89 (1.22)	2.93 (1.23)	2.94 (1.20)	2.56 (1.16)
**Injury**	2.24 (1.31)	2.25 (1.30)	2.14 (1.31)	2.38 (1.34)

Note: All values are reported on the 5-point Likert scale. Numbers in parentheses represent ± standard deviation. Space refers to respondents’ consciousness about their living space. A higher value means more concern that their living conditions are not spacious enough and are harmful to their (or their children’s) vision.

**Table 4:** Regression analysis of factors that explain the severity in myopia ^†^.

	(1)	(2)	(3)
	All Children	Primary School	Secondary School
Length (years)	0.13 ***	0.15 ***	0.10 ***
	(8.12)	(9.38)	(4.07)
Boy	0.01	0.08 **	0.05
	(1.20)	(1.77)	(0.56)
Growth in Height (cm)	0.01	0.02 *	0.01
	(1.35)	(1.87)	(0.35)
BMI	0.02 *	0.03 **	0.02
	(1.63)	(2.00)	(0.69)
Weight (kg)	−0.01	−0.01 *	−0.00
	(−1.40)	(−1.74)	(−0.49)
Space	−0.03 **	−0.04 **	0.01
	(−1.84)	(−2.05)	(0.37)
Income	0.00	−0.10	0.14 *
	(0.04)	(−1.44)	(1.73)
Mydriatic	0.06 *	0.10 **	0.08
	(1.83)	(1.84)	(0.79)
Eye Care Knowledge	−0.004 **	−0.003 **	−0.01
	(−1.89)	(−1.76)	(−1.09)
Genetic	0.08 *	0.05 *	0.003
	(1.72)	(1.68)	(0.04)
Private School	−0.11 **	−0.11 **	−0.11
	(−2.06)	(−1.90)	(−1.05)
School Performance	−0.02 *	−0.03 ***	0.013
	(−1.80)	(−2.74)	(0.57)
Food Supplement			
Vitamin	−0.01	0.14 *	−0.23 **
	(−0.07)	(1.87)	(−2.15)
Fish oil	−0.02	−0.08	−0.04
	(−0.21)	(−0.69)	(−0.18)
Activity Hours			
Cram School	0.02 *	0.02	0.01
	(1.63)	(1.11)	(0.18)
Electronics	−0.01	−0.04	−0.003
	(−0.65)	(−1.37)	(−0.11)
TV	−0.01	−0.01	−0.02
	(−0.59)	(−0.36)	(−0.57)
Study	0.003	−0.05 *	0.05
	(0.14)	(−1.88)	(1.20)
Sleep	−0.007	0.001	−0.04
	(−0.31)	(0.05)	(−0.94)
Ourdoor Exercise	−0.01 *	−0.003	−0.01
	(−1.60)	(−0.60)	(−1.09)
Middle School	0.243 ***		
	(3.32)		
High School	0.0934		
	(0.72)		
N	522	272	250
R2	0.35	0.43	0.27

^†^ Myopia is measured in absolute value of natural logarithm of the unit of refractive error (D) multiplied by 100. ^‡^ Income is in natural logarithm. Space refers to respondents’ consciousness about their living space, measured on the 5-point Likert scale. A higher value means more concern that their living conditions are not spacious enough and are harmful to their (or their children’s) vision. Length refers to years since the diagnosis of myopia. Numbers shown in the table are regression coefficients with t-values in parentheses. * *p* < 0.1, ** *p* < 0.05, *** *p* < 0.01.

**Table 5:** Regression analysis of factors that explain the progression of myopia ^†^.

	(1)	(2)	(3)
	All Children	Primary School	Secondary School
Length (years)	0.02 *	0.02 **	0.01
	(1.76)	(1.81)	(0.74)
Boy	0.03	0.04 *	0.05
	(1.22)	(1.75)	(0.67)
Growth in Height	0.01 **	0.03 ***	−0.002
(cm)	(1.92)	(3.71)	(−0.16)
BMI	0.02 **	0.02 **	0.003
	(2.28)	(2.00)	(0.17)
Weight (kg)	−0.01 ***	−0.01 **	−0.004
	(−3.37)	(−2.30)	(−0.91)
Space	−0.002	0.01	−0.01
	(−0.22)	(0.60)	(−0.49)
Income ‡	0.03	0.03	−0.001
	(0.76)	(0.94)	(−0.02)
Mydratic Use	−0.03	−0.03	−0.03
	(−1.00)	(−0.66)	(−0.47)
Private School	−0.03	−0.04	0.01
	(−0.85)	(−1.14)	(0.11)
Eye Care	−0.001	−0.0001	−0.001
Knowledge	(−0.32)	(−0.07)	(−0.58)
Genetics	0.03 *	0.06 **	−0.01
	(1.66)	(1.99)	(−0.22)
School Performance	−0.01	−0.01	0.003
	(−0.87)	(−1.26)	(0.17)
Food Supplements			
Vitamin	−0.01	−0.03	0.05
	(−0.12)	(−0.70)	(0.40)
Fish oil	0.01	0.04	−0.02
	(0.28)	(0.73)	(−0.28)
Activity Hours			
Cram School	0.01 *	0.01	0.02
	(1.62)	(1.47)	(1.37)
Electronics	0.04 **	0.01	0.05 *
	(2.26)	(0.45)	(1.80)
TV	−0.01	0.02	−0.02
	(−0.42)	(1.10)	(−0.88)
Study	0.01	−0.02	0.01
	(0.34)	(−0.88)	(0.34)
Sleep	−0.00	0.01	−0.02
	(−0.01)	(0.39)	(−0.73)
Outdoore Exercise	−0.0002	−0.01 *	−0.01
	(−0.04)	(−1.65)	(−0.98)
Middle School	0.002		
	(0.05)		
High School	−0.04		
	(−0.45)		
*N*	522	272	250
R2	0.08	0.15	0.08

^†^ Myopia is measured in absolute value of natural logarithm of the unit of refractive error (D) multiplied by 100. ^‡^ Income is in natural logarithm. Space refers to respondents’ consciousness about their living space, measured on the 5-point Likert scale. A higher value means more concern that their living conditions are not spacious enough and are harmful to their (or their children’s) vision. Length refers to years since the diagnosis of myopia. Numbers shown in the table are regression coefficients with t-values in parentheses. * *p* < 0.1, ** *p* < 0.05, *** *p* < 0.01.
